# Isolation of a *Methanobrevibacter gottschalkii* strain from an Eastern Gray Kangaroo

**DOI:** 10.3389/fmicb.2024.1483533

**Published:** 2024-12-18

**Authors:** James G. Volmer, Paul N. Evans, Rochelle M. Soo, Philip Hugenholtz, Gene W. Tyson, Mark Morrison

**Affiliations:** ^1^Centre for Microbiome Research, School of Biomedical Sciences, Queensland University of Technology (QUT), Translational Research Institute, Brisbane, QLD, Australia; ^2^Frazer Institute, Faculty of Medicine, University of Queensland, Brisbane, QLD, Australia; ^3^School of Chemistry and Molecular Biosciences, Australian Centre for Ecogenomics, The University of Queensland, Brisbane, QLD, Australia

**Keywords:** methanogen, archaea, marsupial, *Methanobrevibacter*, methane

## Abstract

Methanogenic archaea are a group of microorganisms found in the gastrointestinal tract of various herbivores and humans; however, the quantity (intensity) of methane emissions during feed digestion varies. Macropodids, such as the Eastern Gray Kangaroo (*Macropus giganteus*), are considered to be low methane-emitting animals, but their gut methanogenic archaea remain poorly characterized. Characterizing methanogens from animals with low methane emissions offers the potential to develop strategies and interventions that reduce methane emissions from livestock. In this study, we describe a novel strain of *Methanobrevibacter gottschalkii* (EGK), the first *Methanobrevibacter* isolate from a marsupial host. Comparative analyses with other *M. gottschalkii* genomes revealed a high degree of gene conservation, along with strain-specific differences in genes related to membrane transport, xenobiotic metabolism, nucleotide metabolism, and the metabolism of cofactors and vitamins. Notably, the *M. gottschalkii* EGK genome contains multiple copies of large proviral elements, likely acquired through integration events in this strain. *M. gottschalkii* EGK is the first isolated representative of *Methanobrevibacter* from a low methane-emitting animal, providing a valuable reference genome to identify metabolic targets for methane mitigation.

## Background

With growing evidence suggesting long-term detrimental effects of climate change, increasing attention is being directed toward understanding and mitigating anthropogenic contributions to greenhouse gas emissions (IPCC, 2021; Hansen and Stone, 2016). Methane is considered a potent greenhouse gas, with >80 times the global warming potential of carbon dioxide over a 20-year timespan (Forster et al., 2021). Livestock production systems are considered to be a major contributor to methane emissions and represent a critical control point for mitigation (Grossi et al., 2018). Simultaneously, global demand for food and animal products is rising, particularly in regions experiencing improved socioeconomic conditions (Fanzo et al., 2020; Kc et al., 2018; Foley et al., 2011).

Given these dynamics, reducing livestock methane emissions has become imperative from social, environmental, and economic perspectives. However, due to the relatively short half-life of methane in the atmosphere, it has the potential to offer rapid reductions in global warming potential.

Methane emissions from livestock are primarily due to the presence and activity of methanogenic archaea, which are part of the microbial consortia involved in feed digestion. Due to their herbivorous diet and gastrointestinal structure, ruminant animals tend to support the retention of a diverse methanogen community and, compared to other animals, produce relatively large amounts of methane during digestion (Clauss et al., 2020). Many years of research have led to the development and evaluation of various methane mitigation strategies, as recently outlined by Volmer et al. (2023a). However, there remains a critical need to identify and develop additional scalable approaches to further reduce livestock methane emissions.

The genus *Methanobrevibacter* is the most prevalent and abundant lineage of methanogenic archaea in vertebrate animals, including humans (Borrel et al., 2020). Specifically, in ruminants, *M. ruminantium* and *M. gottschalkii* are the predominant species (Danielsson et al., 2017; Malik et al., 2021). Interestingly, several studies have shown a correlation between *M. gottschalkii* prevalence and greater amounts of methane emissions from cattle and sheep (Danielsson et al., 2012, 2017; Shi et al., 2014; Tapio et al., 2017). In contrast, lineages of *M. gottschalkii* have also been identified from macropodids such as kangaroos and wallabies (Evans, 2011; Evans et al., 2009; Hoedt et al., 2016), which are considered to be low-methane emitting animals (Vendl et al., 2015; Von Engelhardt et al., 1978). Therefore, comparing *M. gottschalkii* strains isolated from high and low-methane emitting animals may provide valuable insights into unique, host-specific features. For this purpose, we isolated a novel strain of *M. gottschalkii* from an Eastern Gray Kangaroo (*Macropus giganteus*). Furthermore, we aim to provide a culture- and genome-based characterization of the isolate and comparison with *M. gottschalkii* genomes from other animal hosts, including ruminants.

## Methods

### Isolation of *Methanobrevibacter gottschalkii* EGK

The methanogen isolate EGK was recovered from a fecal sample of an Eastern Gray Kangaroo (*Macropus giganteus*), as described by Volmer et al. (2023b). Briefly, the culture of EGK fecal microbiota with the highest methane concentration was used to inoculate BRN-RF10 medium containing 1% (v/v) methanol (Sigma-Aldrich; 179337) and 1% (v/v) ethanol (Sigma-Aldrich; E7023). Following inoculation, the mixture was pressurized to 150 kPa using a gas mixture of H_2_:CO_2_ in a 80:20 ratio. Streptomycin (600 μg/ml) and ampicillin (200 μg/ml) were also added to this culture to suppress bacterial growth, and the culture was incubated at 37°C with gentle shaking (100 rpm). After several rounds of 10-fold serial dilution in the same media with antibiotics, the resultant culture was confirmed to be bacteria-free through polymerase chain reaction (PCR) (27F/1492R) (Enticknap et al., 2006).

The bacteria-free enrichment was serially diluted 10-fold to extinction, and the highest dilution showing growth was selected for further analysis. Archaeal-specific 16S rRNA PCR (86F/1492R) (Wright and Pimm, 2003) was used to determine an initial taxonomic classification of the archaea, according to the methodology described by Volmer et al. (2023b). Archaeal amplicons were prepared following the gel extraction protocol of the Wizard SV Gel and PCR Clean-Up System, according to the manufacturer's instructions. They were then Sanger sequenced at the Australian Genome Research Facility (AGRF; https://www.agrf.org.au/). The archaeal enrichment was then inoculated onto anaerobic BRN-RF10 agar (1.5% w/v) in an anaerobic chamber (Coy Laboratory Products, MI, USA) with an atmosphere of CO_2_:H_2_:N_2_ in the ratio of 15:5:80, respectively. These agar plates were incubated for 4 weeks at 37°C. After incubation, individual colonies were then selected and propagated using BRN-RF10 broth medium. For cryopreservation, the cultures were subsampled and diluted 1:1 dilution with a sterile, anaerobically prepared 30% (v/v) glycerol buffer, as described by Teh et al. (2021).

### Whole-genome sequencing

The biomass of *M. gottschalkii* strain EGK was harvested from 100 ml of BRN-RF10 culture with a headspace of 150 kPa of H_2_:CO_2_ (80:20) by centrifugation at 15,000 × *g* for 5 min. Both the DNA extraction and its preparation for whole genome sequencing followed the protocols routinely used by the Australian Center for Ecogenomics (https://www.ecogenomic.org). Approximately 200 mg of biomass was transferred into a tube containing ~0.2 g of 0.1 mm glass beads (BioSpec Products #11079101) and combined with 750 μl of Bead Solution (Qiagen #12855-100-BS), along with 60 μl of solution C1. This mixture was then vortexed. The tubes were then heated at 65°C for 10 min and subjected to bead beating at 1,000 × *g* for 5 min using a Powerlyser 24 homogenizer (Mo-Bio #13155). After centrifugation at 10,000 × g for 1 min, the resulting lysate was then extracted using the Qiagen DNeasy Powersoil Kit (cat #12888-100), with a final elution volume of 50 μl. The sample library was prepared, and QC was performed as described, with the addition of the epMotion (Eppendorf # 5075000301) automated platform for preparation and clean-up. As per the manufacturer's protocol, the library was sequenced using NovaSeq6000 (Illumina) with NovaSeq6000 SP kit v1.5, 2 × 150 bp paired-end chemistry. Sequenced samples were trimmed using Trimmomatic (v0.32) (Bolger et al., 2014) and assembled using Spades (v3.14.1) (Nurk et al., 2017), with contigs <1,000 bps removed. Contigs identified by the NCBI contamination screen were also removed during WGS submission. Contig 17 and 24, which contained prophage-associated genes, were removed from the genome by the NCBI contamination screen and thus have been provided as Supplementary material. The estimated completeness and contamination of each genome assembly were determined using CheckM1 (v1.1.2) (Parks et al., 2015) and CheckM2 (Chklovski et al., 2023). CheckM2 was also used to determine general genome statistics. Taxonomic classification was performed using GTDB-Tk release 214 (v2.3.0) (Chaumeil et al., 2019).

### Microscopy and transmission electron microscopy

The samples were heat-fixed on glass microscopy slides and stained using the standard Gram staining technique. The Gram status was determined by visualization using a Nikon Eclipse 50i under 100 × magnification. Transmission electron microscopy (TEM) of EGK was conducted by Dr. Rick Webb at the University of Queensland Center for Microscopy and Microanalyses (https://cmm.centre.uq.edu.au/), as described by Volmer et al. (2023b). Wet mount slides of each culture were visualized using a Zeiss AX10 epifluorescence microscope at 420 nm with a cyan (47 HE) filter set to observe autofluorescence.

### Average nucleotide identity and phylogenetic analyses

Representative strains of *Methanobrevibacter* and other closely related species were identified using the Genome Taxonomy Database (https://gtdb.ecogenomic.org) and downloaded from the NCBI genome database. Average nucleotide identity (ANI) was calculated using fastANI (Jain et al., 2018) and visualized via heatmaps (v1.0.12) in RStudio (2023.12.0). Concatenated archaeal marker gene files were produced using GTDB-Tk release 214 (v2.3.0) (Chaumeil et al., 2019). Phylogeny was determined using IQ-Tree (v2.2.6) with 1000 bootstrap replications (Kalyaanamoorthy et al., 2017; Hoang et al., 2017; Nguyen et al., 2014) and visualized using IToL (Letunic and Bork, 2021).

### Comparative genomics analyses of *M. gottschalkii*

Comparative genomic analyses were performed on *M. gottschalkii* DSM11977, DSM11978, EGK, and *M*. sp. A27. Core and pan-genome analysis was performed using Bacterial Pan-Genome Analysis software (BPGA; v1.3). Comparative analyses of core, accessory, and unique genes were conducted in BPGA using USEARCH (v11.0.667) with a 0.8 similarity threshold. Core genes were defined as being present in all genomes, accessory genes were encoded in multiple genomes, and unique genes were encoded by a single genome. All graphs were visualized in GraphPad Prism (v10.1.0). A further in-depth analysis of differential genes was conducted using DRAM (v1.4.6) (Shaffer et al., 2020). Identified KOs were manually assigned to respective KEGG categories. DRAM annotations were also used to identify tRNAs and rRNAs for each genome. The progressiveMAUVE option in MAUVE (v2015-02-26) (Darling et al., 2004) was used to align *M. gottschalkii* genomes to determine pairwise genome synteny. The genomes were re-ordered according to DSM11977 as it contained the fewest contigs.

## Results

### Isolation and purification of *Methanobrevibacter gottschalkii* strain EGK

Anaerobically prepared methanogen liquid medium (Volmer et al., 2023b) was inoculated with fecal biomass from various Australian marsupials as part of an effort to study the methanogen populations of native Australian herbivores (Volmer et al., 2023b). After 24 h, cultures with the highest methane positivity from wombat, mahogany glider, and Eastern Gray kangaroo samples were subcultured into fresh media with supplementation of different carbon sources for methanogenesis, as described by Volmer et al. (2023b), and antibiotics to suppress bacterial growth. For the Eastern Gray Kangaroo, the enrichment culture supplemented with methanol, ethanol, CO_2_, and H_2_ produced the greatest yield, as measured by optical density and methane production.

Microscopic examination of this culture with UV transillumination confirmed the predominance of autofluorescent rod-shaped microbes. This culture was subsequently serially diluted, and the highest dilution supporting microbial growth after incubation was sampled. PCR confirmed the sample to be archaeal-positive and bacterial-negative. The 16S rRNA amplicon sequences produced from this culture had >98% identity to *Methanobrevibacter gottschalkii* porcine strain PG (98.87% identity) and equine strain HO (98.3% identity) reference sequences (Miller and Lin, 2002).

**Figure 1:**
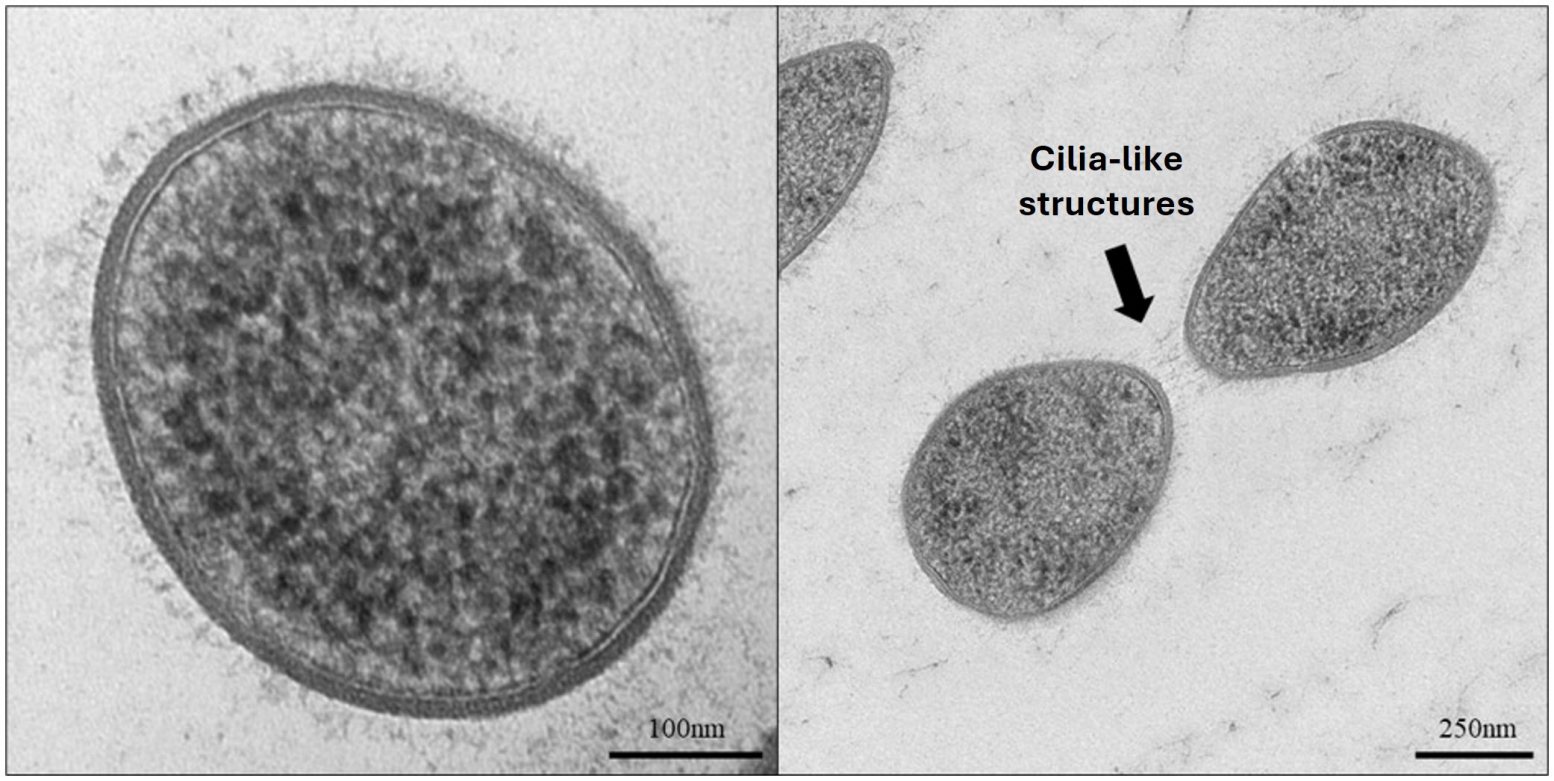
Transmission electron micrographs of *Methanobrevibacter* isolate EGK. The **left panel** shows a simple cell membrane with cilia-like structures. The **right panel** shows two cells potentially interacting via the cilia-like structures.

*Methanobrevibacter gottschalkii* strain EGK was confirmed to be a Gram-positive staining coccobacillus, measuring ~0.5–1.5 μm in length. Transmission electron microscopy (TEM) of strain EGK cells revealed that the archaeon possesses a singular membrane with regular short cilia-like structures covering the cell surface that may facilitate cell–cell interactions (Figure 1).

### Genomic characterization of *M. gottschalkii* strain EGK

The draft genome for *M. gottschalkii* strain EGK and the related strains DSM11977 and 11978, and sp. A27 lacked some rRNA genes but were assessed to be of high quality (Bowers et al., 2017) based on their predicted completeness and contamination scores, as well as the recovery of tRNAs (Table 1). Interestingly, the genome size and number of predicted coding sequences are greatest for strain EGK, and its genome was also found to possess a substantially larger total number of predicted tRNAs.

**Table 1:** Table of genome statistics for *Methanobrevibacter gottschalkii* isolates.

	**DSM11977**	**DSM11978**	**EGK**	**Sp. A27**
CheckM1 completeness	100	100	100	96.8
CheckM1 contamination	0	0	0.4	0
CheckM2 completeness	99.98	99.99	99.99	99.76
CheckM2 contamination	0.2	0.18	0.37	0.14
Coding density (%)	87.8	88.2	88.0	88.3
Contig N50	595,922	121,385	128,749	43,832
Genome size (bps)	1,878,029	1,864,477	1,994,976	1,809,869
GC content (%)	30	30	31	30
Coding sequences	1,848	1,897	2,035	1,801
Total contigs	6	20	49	58
Total no. tRNAs	33	35	58	29
Canonical tRNAs (/20)	20	20	20	18
No. 5S rRNA	0	0	0	0
No. 16S rRNA	1	2	0	0
No. 23S rRNA	1	1	1	1

CheckM1 and CheckM2 values for completeness and contamination have been displayed, respectively. General genome statistics were determined using CheckM2. The number of tRNAs and rRNAs was determined using DRAM.

**Figure 2:**
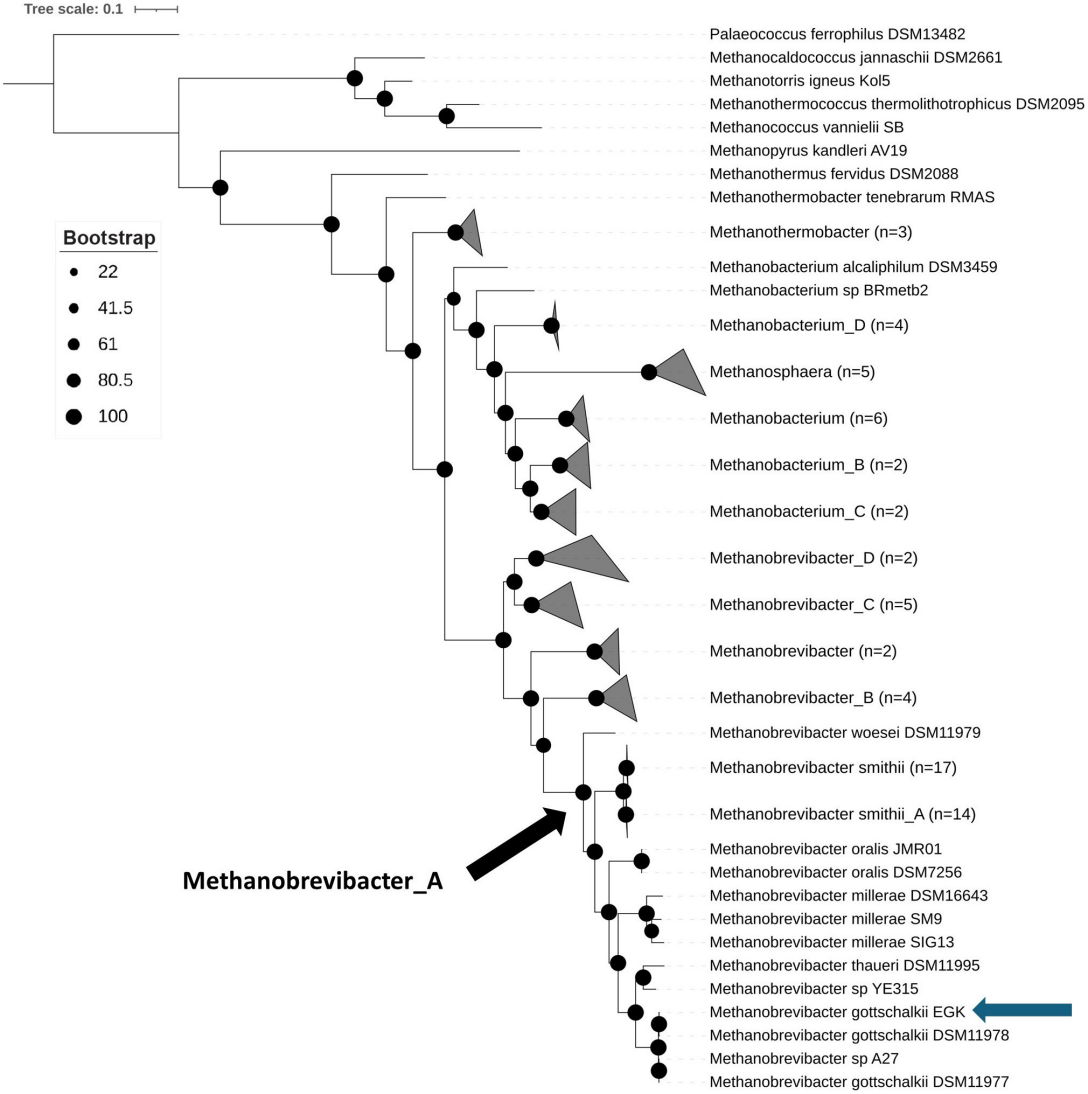
Phylogenetic tree showing isolate EGK to be a novel strain of *Methanobrevibacter gottschalkii*. Concatenated archaeal marker genes were produced by GTDB-Tk and phylogeny inferring using IQ-Tree with 1,000 bootstrap replications. The subsequent phylogenetic tree was visualized using IToL with *Palaeococcus ferrophilus* DSM13482 used as the outgroup. Bootstrap values are displayed by the black circles, as per the legend. The blue arrow indicates the position of isolate EGK. The black arrow shows the last common ancestor of *Methanobrevibacter*_A.

Concatenated archaeal marker genes from *M. gottschalkii* EGK and selected Methanobacteriota reference genomes were identified using GTDB-Tk, and phylogeny was inferred using IQ-Tree (Figure 2). The resulting phylogenetic tree confirms that strain EGK represents a novel isolate of *Methanobrevibacter gottschalkii* within the genus *Methanobrevibacter*_A, alongside *Methanobrevibacter smithii* and *M. smithii_*A.

This finding was further validated by pairwise average nucleotide identity (ANI) analysis, which showed that EGK forms a distinct cluster with other *M. gottschalkii* genomes, sharing >99% ANI, well above the 95% ANI threshold used for species demarcation (Supplementary Table S2) (Richter and Rosselló-Móra, 2009). The deep phylogenetic divergence of *Methanobrevibacter_*A, supported by relative evolutionary divergence values from GTDB, suggests that *Methanobrevibacter*_A likely represents a unique genus separate from *Methanobrevibacter*, suggesting that the type strain of *Methanobrevibacter, M. ruminantium*, belongs to a distinct genus from *M. gottschalkii*. Moreover, as *M. ruminantium* and *M. gottschalkii* are associated with different methane-emitting phenotypes, this distinction highlights important phylogenetic and biological differences between these dominant ruminant methanogens. Therefore, we propose that *Methanobrevibacter*_A be reclassified as *Methanoenterobius* to better reflect the host-associated nature of species within this genus.

### Comparative analyses of *M. gottschalkii* genomes

**Figure 3:**
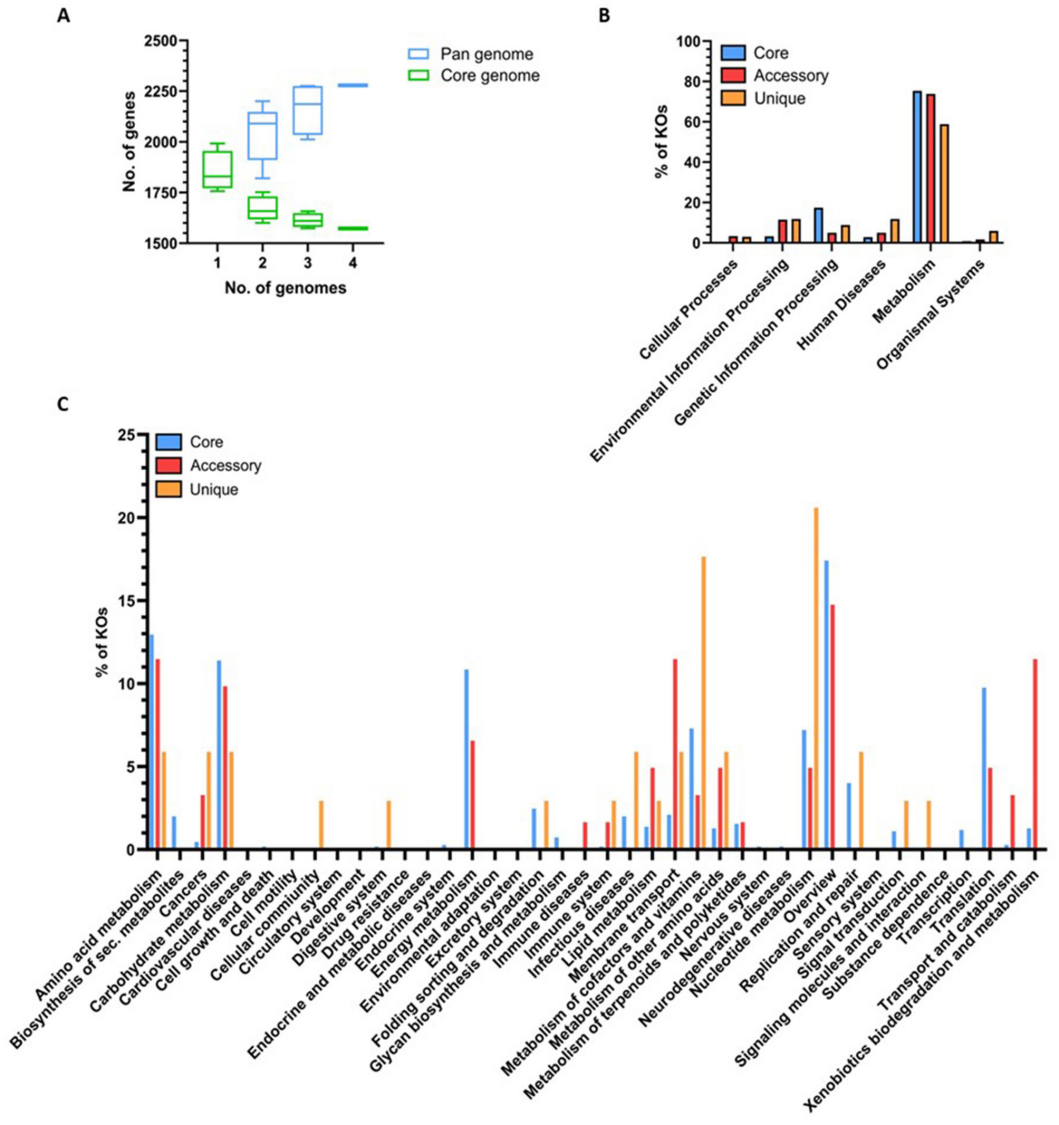
Comparison of predicted coding capacity and KO annotation between strains. Pan-core gene counts and KO annotations were determined using BPGA (v1.3) and visualized in GraphPad Prism. **(A)** Accumulative number of genes in the core and pan-genome with the increasing number of *Methanobrevibacter gottschalkii* genomes. **(B)** Number of *M. gottschalkii* genes assigned to KOs within the six high-level KEGG categories. **(C)** Number of *M. gottschalkii* genes assigned to KOs within second-level KEGG categories.

Based on the results presented in Figures 2, 3, the genomes of the four *M. gottschalkii* strains were further compared, with the “core” genome of 1,573 genes representing ~83% of the total gene counts (Figure 3A). Most of the genes that could be confidently assigned to KOs within the core and non-core (i.e., accessory and unique) components of the pan-genome were associated with metabolism (Figure 3B). Further categorization of core genes identified 22 categories, with the most populated being “general overview” (17.4%), “amino acid metabolism” (12.9%), “carbohydrate metabolism” (11.4%), “energy metabolism” (10.8%), and “translation” (9.8%; Figure 3C). Similarly, genes with KO classifications from the non-core were assigned to many of the same subcategories, although at greater proportions than core genes for membrane transport (11.5%) and xenobiotic biodegradation and metabolism (11.5%). KOs exclusively encoded by one of the four genomes were mostly assigned to nucleotide metabolism (20.6%) and metabolism of cofactors and vitamins (17.6%). These differences in the non-core components are most likely adaptations to the variations in the nutritional and/or physiological ecology of the different hosts.

**Figure 4:**
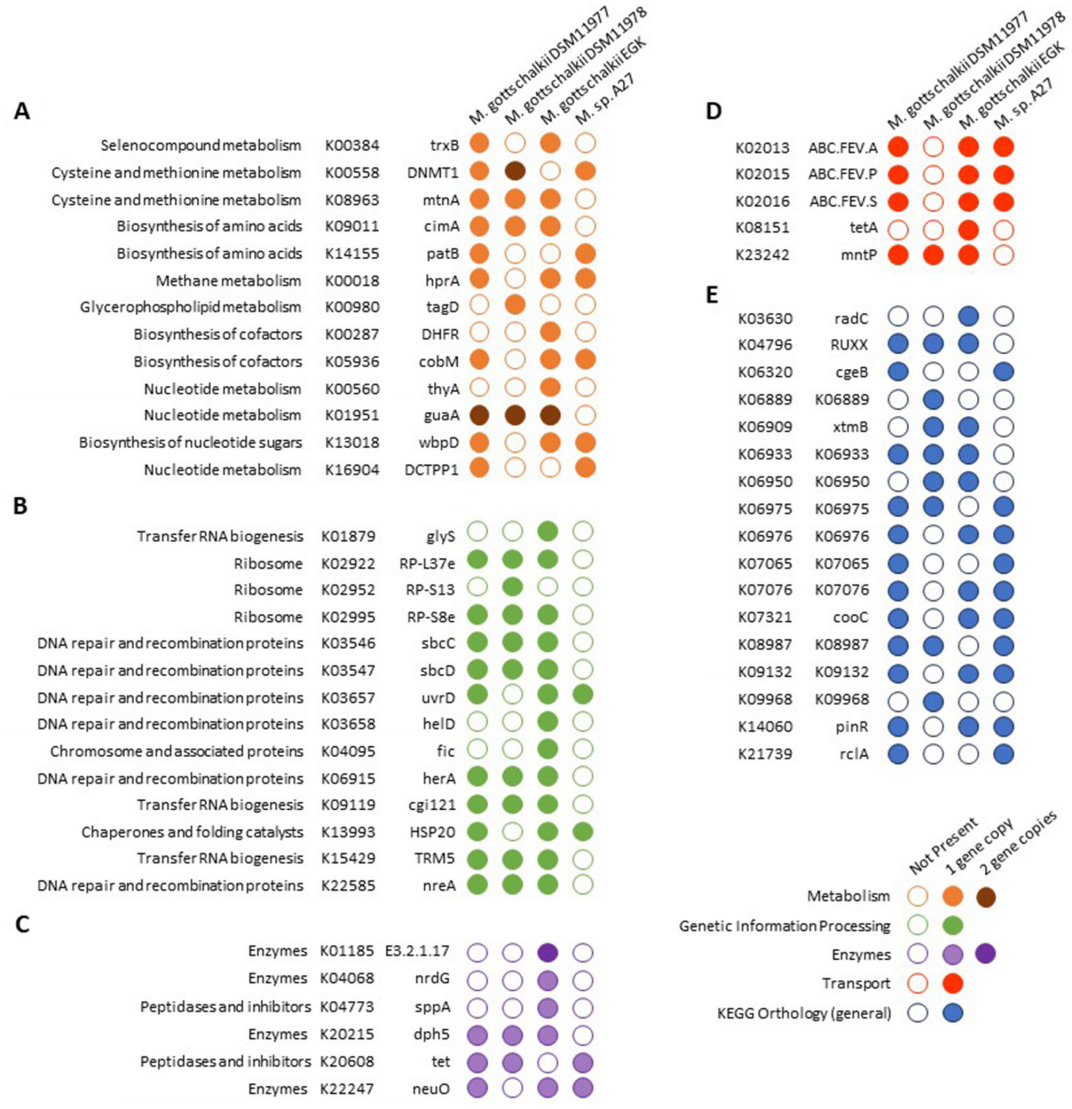
Comparative analyses of KEGG gene annotations between *Methanobrevibacter gottschalkii* genomes. KEGG Orthologs (KOs) were annotated using DRAM. Only genes that were present/absent from a given genome were included in the analyses. Genes were only assigned to a single category. **(A)** Metabolism, **(B)** genetic information processing, **(C)** enzymes, **(D)** transporters, **(E)** and KEGG orthology (general).

Methanogenesis-associated genes were highly conserved between the strains, suggesting that all strains can perform hydrogenotrophic methanogenesis using CO_2_/H_2_ and formate. The only differentially encoded KO potentially involved in methane metabolism was glycerate dehydrogenase (hprA; K00018; Figure 4A), which was not encoded by DSM11978. Most differentially enriched KOs associated with genetic information processing were encoded by EGK and enriched against *M*. sp. A27, with DNA helicase IV (helD, K03658) and cell filamentation protein fic (K04095) encoded by EGK (Figure 4B). Three ABC transporters encoding for iron transport (K02013, K02015, and K02016) were present in all strains except *M. gottschalkii* DSM11978 (Figure 4D).

Additional uncharacterized KOs (Figure 4E) and numerous genes without KO annotations were differentially enriched, requiring further functional investigation to determine their effect on the phenotypic characteristics of each strain.

### *M. gottschalkii* EGK encodes virally acquired genes

**Figure 5:**
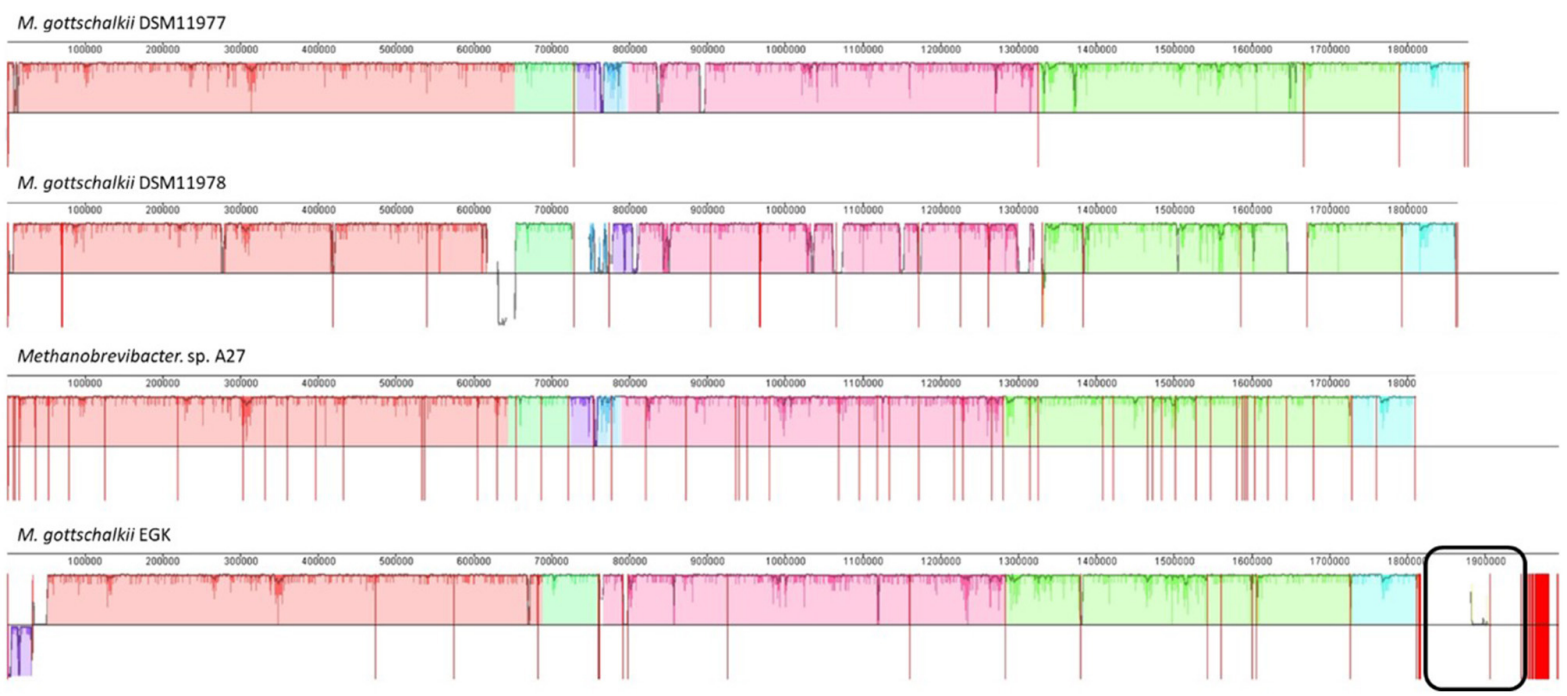
Multiple genome alignment of EGK with other *Methanobrevibacter gottschalkii*. The genomes were aligned using the progressiveMAUVE function in MAUVE, with all genomes reordered according to *M. gottschalkii* DSM11977, given it had the fewest number of contigs. The genomes display high synteny, with unique regions observed in EGK and DSM11978. The black box shows the two large putative viral contigs of EGK.

Given the larger genome size of *M. gottschalkii* EGK and its unusual animal host, further comparative analyses were performed to identify any strain-specific genomic characteristics. Sequence alignment of the *M. gottschalkii* strains showed high synteny among the genomes, which is expected given that they represent different strains of the same species. However, both DSM11978 and EGK contain multiple strain-specific regions. EGK contains two contigs (~88 and ~40 Kbp, respectively), with only one of the contigs containing a small region of synteny with DSM11978 (Figure 5). These regions contain many predicted tRNAs, which may explain the larger total numbers compared to the other strains (Table 1). In total, 92% (117/127) and 97% (43/44) of the genes for the two EGK contigs were hypothetical. The only annotated genes in these regions were predicted phage proteins (head, baseplate, and tail proteins), endonucleases, phage lysozymes, and invasins, suggesting that EGK may have acquired these genes through prophage integration, and DSM11978 may contain a prophage from a different acquisition event.

Further comparison of these genes showed EGK encoded for dihydrofolate reductase (K00287) and thymidylate synthase (K00560), along with two copies of lysozyme (K01185), anaerobic ribonucleoside-triphosphate reductase activating protein (K04068), and protease IV (K04773; Figure 4C), all located in the ~80Kbp region identified (Figure 4). Tetracycline resistance protein tetA (K08151) and a putative prophage DNA-invertase (K14060) were additionally encoded by EGK (Figures 4D, E). These genes were also encoded on a ~10 Kbps contig along with phage and virus-associated proteins, suggesting that this contig may represent additional viral genes.

## Discussion

*Methanobrevibacter gottschalkii* has been identified in macropodids using cultivation-independent approaches (Vendl et al., 2015; Von Engelhardt et al., 1978), and the *Methanobrevibacter* sp. isolate WBY1 from a Tammar Wallaby (*Notamacropus eugenii*) was confirmed to be closely related to *M. gottschalkii* by 16S rRNA gene analysis (Evans et al., 2009) before viable cultures of this strain were lost. As such, *M. gottschalkii* strain EGK is the sole isolate from Macropodidae, a lineage of marsupial herbivores with a distinctive adaptation of their digestive anatomy to support pre-gastric (foregut) hydrolysis of plant biomass via a complex community of microbes. To the best of our knowledge, since the first isolation of *M. gottschalkii* from porcine and equine samples (Miller and Lin, 2002), no other cultured isolates have been made publicly available, despite a high prevalence of the species in the rumen microbiome (Volmer et al., 2023a). As such, strain EGK represents one of the few isolates of the species, recovered from an animal with unique host physiology.

Similar to many other animals, the gut microbiota of marsupial herbivores is involved in the digestion of plant biomass but with relatively small amounts of methane emissions compared to domesticated ruminant species (Vendl et al., 2015; Von Engelhardt et al., 1978). However, *M. gottschalkii* represents one of the most dominant and prevalent *Methanobrevibacter* species in ruminant animals (Danielsson et al., 2017; Malik et al., 2021), and several studies have shown a link between *M. gottschalkii* and greater methane production in sheep and cattle (Danielsson et al., 2012, 2017; Shi et al., 2014). In this study, we showed that the genomic content of the four cultured isolates of *M. gottschalkii* is highly conserved, with >85% of their coding sequences shared among all four strains and with ANI >98%. However, some genes of interest involved in methanogenesis, nutrient uptake, and metabolism are unique to each species. Glycerate dehydrogenase was encoded by all strains except DSM11978, has been linked to serine cycling in methylotrophic bacteria, and is required for C_1_ metabolism (Chistoserdova and Lidstrom, 1994). DSM11978 also did not encode predicted ABC transporters for iron, with differential enrichment of trace metal ABC transporters observed across other species of marsupial methanogens (Volmer et al., 2023b). Furthermore, differentiations in genes encoding for predicted xenobiotic degradation and metabolism suggest that the strains may have adapted to help break down potentially harmful compounds in their host environment. Typically, xenobiotic degradation is associated with environmental methanogens, which can either directly metabolize compounds or maintain thermodynamics by syntrophic cooperation with other bacteria (Schink, 1997; Junghare et al., 2019). Native Australian marsupials are known to have unique folivore diets, rich plant terpenes, and phenolic compounds (Stupans et al., 2001), to which the methanogens may have adapted with a resultant metabolic burden and comparatively slower methanogenesis, though this requires experimental validation. Overall, these differentially encoded genes likely represent a strain-specific adaptation to their respective host environment and may directly or indirectly affect the methane production of the host animals.

The most apparent difference between the isolates was the presence of multiple large regions in the genome of strain EGK containing several phage-associated and integrase genes. This finding provides further evidence that members of this genus encode proviral elements, including *M. gottschalkii* DSM11978, *M. millerae, M. ruminantium, M. olleyae, M. smithii, M. oralis*, and *M*. *wolinii* (Medvedeva et al., 2023). Interestingly, the prophage identified in the EGK genome shows limited similarity to that from DSM11978, suggesting that the prophage of EGK was uniquely acquired by this strain. Recently, Baquero et al. (2024) showed the stable coexistence of a temperate archaeal virus (MSTV1) in the human methanogen *Methanobrevibacter smithii*, with changes in gastrointestinal environment and metabolites inducing viral production and release. Similarly, EGK may encode for prophase, which could be used to modulate EGK itself or the wider microbiome, given that this novel genome region also encodes for predicted lysozymes, proteases, and tetracycline resistance.

## Conclusion

The isolation of *M. gottschalkii* EGK further expands the limited number of cultured methanogen isolates, specifically those recovered from low methane-emitting animal hosts, and demonstrates that this strain is homologous to those from high-methane-emitting animals with some notable genetic differences. Given that *M. gottschalkii* is typically associated with high methane production in ruminants, this newly cultured isolate provides an essential resource for further analyses of factors affecting methane production. Despite being highly associated with the phenotypes, it is worth noting that there is currently a distinct lack of *M. gottschalkii* in high-methane-emitting animals. Thus, a key focus for future work should be expanding the cultured representatives. The addition of *M. gottschalkii* EGK and other cultured methanogens with strains recovered from high and low-methane emitting hosts allows for future characterization of the contributions that inhabitant methanogen communities provide to high and low-methane phenotypes compared to differing gastrointestinal physiology and environmental factors.

### Description of *Methanoenterobius*

We suggest the reclassification of *Methanobrevibacter*_A to *Methanoenterobius* (Entero-, referring to the intestinal tract of animals; methano-, referring to methane production; and -brevibacter, referring to cellular morphology of the organisms) to identify *Methanobrevibacter*_A as a unique genus and reflect its prevalence in gastrointestinal-associated microbiomes of animals.

## Data Availability

The datasets presented in this study can be found in online repositories. The names of the repository/repositories and accession number(s) can be found at: https://www.ncbi.nlm.nih.gov/, JBANCJ000000000 and PRJNA1074504. The ZooBank accession for ‘Methanoenterobius' is ‘0FAAF0FB-C1FC-436A-B381-CEB907855833'. The ZooBank accession for this article is ‘2B48ED25-2B47-4EB7-AA06-D65BF0E82640'.
